# Fruit and Vegetable Accessibility in the Home: Intervention Changes and Cross-Sectional Associations with Diet Quality

**DOI:** 10.3390/children13040577

**Published:** 2026-04-21

**Authors:** Adriana Verdezoto Alvarado, Shannon M Robson

**Affiliations:** 1 Department of Individual, Family, and Community Education, University of New Mexico, 504 Cornell Dr. NE, Albuquerque, NM 87131, USA; 2 Department of Health Behavior and Nutrition Sciences, University of Delaware, 26 N College Avenue, Newark, DE 19713, USA; robson@udel.edu

**Keywords:** fruit accessibility, vegetable accessibility, home food environment, diet quality

## Abstract

**Highlights:**

**What are the main findings?**

- An 8-week parent-focused intervention targeting fruit and vegetable (FV) accessibility domains of location, form, and visibility significantly changed parents’ perceptions of form (FV ready-to-eat) in the home but did not significantly improve objective FV accessibility, availability, or overall diet quality in children.
- In exploratory analyses, children and parents in the higher diet quality category tended to have greater FV accessibility (location and visibility) in the home. Although some associations reached statistical significance, including higher FV visibility with children’s HEI scores above national averages at post-intervention and FV location (within reach) with parent HEI scores above national averages at pre- and post-intervention, these findings are exploratory and should be interpreted with caution.

**What are the implications of the main findings?**

- These findings suggest that FV accessibility is an important aspect of the home environment, warranting further investigation into how this domain may meaningfully shape intake behaviors.
- FV accessibility appears to be a distinct component of the home food environment, highlighting the need for future research to develop and validate more comprehensive measures that better capture this domain.

**Abstract:**

Background/Objectives: Fruit and vegetable (FV) availability/accessibility are associated with increased intake of FVs and are important determinants of intake. The aim of this analysis was to evaluate the pre-post changes in an FV accessibility intervention and examine cross-sectional associations between accessibility domains and diet quality categories at pre- and post-intervention. Methods: Thirty parent–child dyads (mean age = 41.2 ± 4.7; 9.2 ± 1.9) completed an 8-week pre-post intervention. Assessments included perceptions of accessibility, the Home Food Inventory with added accessibility domains, and three-day diet records used to calculate HEI-2020 scores. Stuart–Maxwell tests were used to evaluate changes in categorical responses, paired *t*-tests assessed pre-post changes, and independent *t*-tests compared accessibility by HEI category. Results: Parents reported a perceived increase in frequency of having the form of FVs prepared/ready for use (*p* = 0.034). No significant pre–post changes were observed in objective FV availability/accessibility domains, FV intake, or HEI scores for children and parents. Exploratory analyses showed that children and parents with HEI-total scores above national averages had higher mean FV location and visibility, with post-intervention visibility being significantly associated with higher HEI among children (*p* = 0.048) and location being significantly associated with higher HEI among parents at pre- (*p* = 0.033) and post-intervention (*p* = 0.046). Conclusions: The FV accessibility intervention did not significantly improve objective HFE accessibility or diet quality in this small sample. Exploratory findings suggest that FV accessibility domains may be associated with diet quality; however, these observations are preliminary. Larger and longer-term studies are needed to determine whether modifying FV accessibility can meaningfully improve children’s dietary intake.

## 1. Introduction

National guidelines recommend following a healthy dietary pattern, as the foods and beverages people consume can have a great impact on their health [1]. The home food environment (HFE) is a setting in which children grow and develop and has been associated with eating behaviors [2]. Within a social-ecological framework, dietary behaviors result from interactions across multiple contexts (e.g., physical and social environments) and multilevel influences spanning individual and social networks [3]. The HFE is a key interpersonal and environmental context in which parents can influence children through food-related practices and exposures by structuring the home to support healthy behaviors.

The physical HFE (defined as availability and accessibility to food) has been associated with higher consumption of fruits and vegetables (FVs) when those are available in the home [4]. As nutrition gatekeepers [5], parents play a key role in shaping the HFE and can influence children’s intake by increasing both the availability and accessibility of FVs through food purchasing, preparation, and placement within the home [5,6]. Despite this, relatively few intervention studies have focused solely on modifying the physical HFE, and even fewer have targeted FV accessibility among children [7]. Accessibility may represent a distinct and actionable intervention target because it extends beyond the presence of food, providing parents with opportunities to present environmental cues that facilitate children’s access to FVs [8].

Even though availability and accessibility are often discussed together, these constructs are conceptually distinct and are not always used consistently throughout studies. Availability refers to the physical presence of foods in the home, whereas accessibility refers to whether available foods are prepared, placed, or presented in ways that facilitate consumption [9,10]. A systematic review examining interventions aimed at increasing FV availability in children’s organizational nutrition environments found that only 3 of 23 studies specifically targeted the home and families, and 1 targeted a school environment but measured changes in the children’s home [7]. Among these four studies, findings were mixed. One study reported a significant intervention-related increase in FV availability [11], two found no significant intervention effect on FV availability [12,13], and two reported no significant changes in FV accessibility [13,14]. Even though findings for FV availability were mixed, all of the studies that assessed knowledge or awareness reported significant improvements, suggesting that educational components were effective in increasing understanding of the role of FVs [7]. Despite this, the extent to which improvements in knowledge translate into measurable changes in the physical HFE, particularly in accessibility, remains unclear.

Interventions targeting FV intake often address multiple domains simultaneously, including food availability, nutrition education, and behavioral strategies [15]. Although these multidomain approaches are valuable, their complexity makes it difficult to determine which aspects of the HFE most strongly influence FV intake and overall diet quality. Accessibility, a behavioral determinant of eating patterns, represents a modifiable domain that can be isolated and examined [16]. By specifically examining accessibility, we can better understand how environmental cues within the home may shape children’s eating behaviors and contribute to diet quality.

Our previously published work demonstrated the feasibility of an accessibility intervention, with strong recruitment, enrollment, and parent satisfaction rates [9,17]. Accessibility was defined as setting FVs in a location (within reach), form (cooked or ready-to-eat), and visible to the child to encourage consumption. The current analysis evaluates the pre-post changes in an FV accessibility intervention delivered to parents of 6–12-year-old children. We also examined cross-sectional associations between accessibility domains (location, form, visibility) and Healthy Eating Index (HEI) by comparing families with HEI-total scores above versus below HEI national averages [18] for parents and children pre- and post-intervention.

## 2. Materials and Methods

This study is an analysis of an intervention previously focused on assessing the feasibility of an FV accessibility intervention [9,17]. As previously reported, the 8-week intervention was delivered virtually to parents and focused on making 2 fruits and 3 vegetables accessible for 6–12-year-old children. The intervention targeted three domains of FV accessibility, defined as foods that are in a location, form, and visible to the child [9] and used weekly goal setting, a behavioral modification strategy from the Social Cognitive Theory [19]. Verbal goal attainment (yes/no) established in previous sessions was evaluated at the beginning of sessions 2 through 8; regardless of attainment, participants proceeded to set new goals for the following week.

Parents were eligible if they were the primary parent responsible for obtaining and preparing food for the child, reported having at least 2 types of fruit and 3 types of vegetables available in the home, and agreed to in-home assessments by research personnel. Thirty-two parents enrolled in the study, and 30 completed the post-assessment, leaving a final sample size of 30 parents and children for this analysis. Parents completed written informed consent, and children provided verbal assent. This study was approved by the University of Delaware Review Board (ID no. 2015683-1).

### 2.1. Measures

All measures were completed at pre- and post-intervention except for the self-reported demographic questionnaire, which was completed at baseline only. Parents self-reported parent/child demographic data (e.g., age, sex, race, and ethnicity) and annual household income. Household food security was verbally assessed by research personnel during the home visits using the U.S. Household Food Security Survey Module: Three-Stage Design with Screeners [20] with a 30-day reference period. Food security raw scores were summed based on a range from 0 to 18, with lower scores indicating high/marginal food security.

Subjective home accessibility of FVs was assessed using questions from Hearn and colleagues [21]. The questions were as follows: “During the past week, how frequently were there fruits or vegetables on the kitchen counter or somewhere in the open week?” “During the past week, how frequently was there ready-to-eat fruit (whole or cut-up) or ready-to-eat vegetables (whole or cut-up) on the front shelf of the refrigerator (that could be visibly seen when the door is open)?” and “During the past week, how frequently were fruits or vegetables in the refrigerator or freezer prepared so they could be readily used?” Response options were most of the time, some of the time, seldom and never. For the analyses, the seldom and never categories were combined.

The Home Food Inventory (HFI) was completed by a trained staff member. The HFI is a validated checklist that assesses food items present or absent (yes/no) in the home environment [22]. The FV availability score, a subcategory score, was derived from the HFI and comprised 26 different fruits and 20 different vegetables [23]. The FV availability score ranged from 0 to 46 (higher scores indicate a greater variety of FVs available). Fruit categories included fresh, canned, frozen and/or dried and vegetable categories included fresh, canned, and/or frozen. The HFI was modified to assess three accessibility domains for each available FV using yes/no responses. These additional accessibility items did not undergo formal psychometric evaluation. Location was defined as whether the child could reach the item (based on the child’s height). Form was defined as whether the FV was ready-to-eat or cooked (for example, cut-up pineapple, baby carrots, or cut-up vegetables). Visibility was defined as whether the FV was visible without moving items, including when a child opens the refrigerator or cabinet. Accessibility scores for location, form, and visibility were summed separately, with higher scores indicating greater accessibility within each domain.

Parent and child height and weight were measured by trained research staff during pre- and post-intervention home visits. Participants wore light clothing (empty pockets) and no shoes. Height was measured in triplicate to the nearest 0.5 cm using a portable stadiometer (Seca 213, Hanover, MD, USA); a fourth measurement was obtained if any two measures differed by more than 0.5 cm. Weight was measured in triplicate to the nearest 0.1 kg using an electronic scale (Seca 874, Hanover, MD, USA). For parents, body mass index (BMI; kg/m^2^) was calculated from the mean weight and height. For children, BMI percentiles were calculated from mean height and weight using the 2010 Centers for Disease Control and Prevention growth charts [24].

Parents completed three days of dietary intake (two weekdays plus one weekend day) for themselves and the child. Days could be recorded consecutively or non-consecutively. Research personnel reviewed diet records and asked clarifying questions about foods or quantities when applicable. Diet records were entered into Nutrition Data Systems Research (NDS-R) 2024 software developed by the Nutrition Coordinating Center, University of Minnesota (Minneapolis, MN, USA). NDSR automatically generates the FV serving count based on the 2000 Dietary Guidelines for Americans (DGAs) [25]. Fruit ½ cup serving equivalents were defined as one medium fruit, ½ cup chopped fresh, frozen, cooked, or canned fruit, or ¼ cup of dried fruit; vegetable ½ cup serving equivalents were defined as 1 cup of raw leafy vegetables or ½ cup of other cooked or raw vegetables. Dietary data across the three days were averaged and analyzed for diet quality using the Healthy Eating Index (HEI)-2020 simple method algorithm provided by the National Cancer Institute [26]. The HEI-2020 aligns with the 2020–2025 DGAs [27]. Total scores range from 0 to 100, with higher scores indicating greater adherence to the DGAs. For exploratory analyses, HEI-total scores were dichotomized into “below” and “above” national averages separately for parents and children. The national average HEI-total score based on the National Health and Nutrition Examination Survey 2017–2018 was 54 for children ages 2–18 and 57 for adults aged 19–59 [18].

### 2.2. Statistical Analysis

Means and standard deviations for continuous variables and frequencies for categorical variables were calculated. Normality of continuous variables was assessed using the Shapiro–Wilk test and inspection of Q-Q plots. Stuart–Maxwell tests were used to determine pre-post changes in subjective categorical responses of parents who reported FV accessibility as seldom/never, some, or most of the time at pre- and post-intervention. To compare pre- and post-intervention changes, paired *t*-tests were used for continuous, normally distributed variables (e.g., HEI scores for parents and children and FV availability), while Wilcoxon signed-rank tests were used to compare variables not normally distributed (including domains of FV accessibility, several FV categories, and servings of FVs). For exploratory analysis, mean accessibility domains (location, form, and visibility) were compared between participants above versus below the national HEI total score reference at each time point (pre- and post-intervention). Independent *t*-tests were used for normally distributed variables, with equal variance assumptions assessed using Levene’s test. The FV accessibility domain of form at week 8 was analyzed using a Mann–Whitney U test due to non-normal distribution. All analyses were conducted using SPSS Version 31.0, SPSS Inc., Chicago, IL, USA. 2025. A *p*-value of <0.05 (two-tailed) was considered statistically significant.

## 3. Results

### 3.1. Participant Characteristics

Thirty parent/child dyad characteristics are presented in Table 1. The mean age of parents was 41.2 ± 4.7, and the mean age for children was 9.2 ± 1.9 years. All participants (100%) in this sample reported high/marginal food security.

### 3.2. Subjective Perceptions of FV Accessibility

The Stuart–Maxwell test found no statistically significant difference between pre- and post-intervention on the frequency of FVs on the kitchen counter or somewhere in the open (*p* = 0.352) or ready-to-eat fruit or vegetables on the front shelf of the refrigerator (*p* = 0.118). There was a statistically significant difference (*p* = 0.034) between pre- and post-intervention for the frequency of FVs in the refrigerator or freezer prepared so they could be readily used, with (*n* = 11) participants moving to a higher category (see Table 2).

### 3.3. Home Food Inventory

The mean summed availability of FVs decreased from pre- (M = 17.03 ± 5.4) to post-intervention (M = 16.17 ± 5.7), but this change was not statistically significant: t(29) = −1.39, *p* = 0.088, d = −0.253. When examined by FV category, a significant decrease in fresh fruit availability was observed from pre- (M = 5.03 ± 2.6) to post-intervention (M = 4.07 ± 2.4; *p* = 0.015, Z = −2.43). No significant changes were observed for frozen fruit (pre: 1.9 ± 1.6; post: 2.2 ± 1.9; *p* = 0.405, Z = −0.833), canned fruit (pre: 1.20 ± 1.2; post = 1.23 ± 1.1; *p* = 0.946, Z = −0.068), or dried fruit (pre: 0.67 ± 0.8; post: 0.90 ± 1.3; *p* = 0.221, Z = −1.22). For vegetables, no significant pre- to post-intervention changes were observed for fresh vegetables (pre: 5.37 ± 2.4; post: 5.13 ± 2.3; t(29) = −0.587, *p* = 0.281, d = −0.107), frozen vegetables (pre: 3.20 ± 1.6; post: 2.73 ± 2.0; *p* = 0.105, Z = −1.62), or canned vegetables (pre: 1.13 ± 1.3; post: 1.23 ± 1.2; *p* = 0.521, Z = −0.642). Minimal changes were seen across each accessibility domain (location, pre: 15.0 ± 5.7; post: 13.5 ± 6.7, *p* = 0.070, Z = −1.81); form (pre: 5.5 ± 4.2; post: 6.5 ± 4.3; *p* = 0.656, Z = −0.445); and visibility (pre: 11.2 ± 4.4; post: 11.4 ± 5.3; *p* = 0.958, Z = −0.052)), though no changes were statistically significant.

### 3.4. Diet Quality and FV Intake

HEI-total scores decreased from pre- to post-intervention for both children and parents. Among children, scores declined from 56.9 ± 10.7 at pre-intervention to 53.7 ± 10.2 at post-intervention, but this change was not statistically significant, t(29) = −1.50, *p* = 0.071, d = −0.275. Parents showed a similar pattern, with HEI-total scores decreasing from 60.4 ± 13.2 to. 57.9 ± 11.2, also not statistically significant, t(29) = −0.98, *p* = 0.167, d = −0.180. No significant changes were observed for servings of FVs consumed by children (pre: 3.06 ± 2.2; post = 3.09 ± 2.4; *p* = 0.861, Z = −0.175) or parents (pre: 4.01 ± 2.4; post = 3.46 ± 2.2; *p* = 0.365, Z = −0.905).

### 3.5. Associations Between Accessibility Domains and Diet Quality

Even though the intervention focused on increasing FV accessibility, exploratory cross-sectional analyses were conducted to examine associations between accessibility domains and diet quality categories. Among children at pre-intervention, no significant differences were observed by HEI-total score category. Children with HEI-total scores below national averages had lower mean scores for FV location (M = 13.09 ± 5.1 vs. M = 16.16 ± 5.9; *p* = 0.081, d = 0.545), form (M = 4.0 ± 2.6 vs. M = 6.37 ± 4.7; *p* = 0.069, d = −0.578), and visibility (M = 9.81 ± 4.3 vs. M = 11.95 ± 4.4; *p* = 0.103, d = −0.490). At post-intervention, a similar pattern was observed, with FV visibility significantly higher among children with HEI-total scores above the national average (M = 13.0 ± 5.7) compared to those below (M = 9.80 ± 4.4, t(28) = −1.723, *p* = 0.048, d = −0.629). Differences for FV location (M = 11.67 ± 5.0 vs. M = 15.27 ± 7.7; *p* = 0.072, d = −0.549) and form (M = 6.27 ± 3.8 vs. M = 6.73 ± 4.9; *p* = 0.388, d = −0.105) were not statistically significant.

Among parents at pre-intervention, FV location was significantly higher for those with HEI-total scores above the national average at baseline (M = 16.71 ± 5.9) compared to those below (M = 12.84 ± 4.8, t(28) = −1.909, *p* = 0.033, d = −0.703). No significant differences were observed for FV form (M = 4.54 ± 3.3 vs. M = 6.23 ± 4.7; *p* = 0.140, d = −0.406) or visibility (M = 9.92 ± 3.8 vs. M = 12.12 ± 4.7; *p* = 0.090, d = −0.507). At post-intervention, FV location remained significantly higher among parents with HEI scores above the national average (M = 15.53 ± 7.4) compared with those below (M = 11.40 ± 5.4, t(28) = −1.750, *p* = 0.046, d = −0.639), while no significant differences were observed for FV form (M = 6.53 ± 5.7 vs. M = 6.47 ± 2.7; *p* = 0.466, d = 0.015) or visibility (M = 9.93 ± 4.6 vs. M = 12.87 ± 5.6; *p* = 0.064, d = −0.572).

## 4. Discussion

This analysis evaluated the pre-post changes in an FV accessibility intervention delivered to parents of 6–12-year-old children and examined cross-sectional exploratory associations between accessibility domains and diet quality. Although statistically significant improvements in objective FV availability, accessibility, and overall diet quality were not observed, changes in subjective perception and the observed cross-sectional patterns provide preliminary insight into accessibility and highlight areas that may be explored in future work.

Subjective accessibility measures of FV preparation increased, with 11 parents reporting that they had FVs in the refrigerator/freezer prepared so they could be readily used. Similarly, a randomized newsletter-based intervention targeting parents and adolescents reported significantly higher parent-reported FV accessibility in the intervention group compared with controls at post-intervention and follow-up when both availability and accessibility were explicitly addressed [28]. Given that parents play an integral role in structuring the HFE and shaping children’s dietary behaviors [29], these findings suggest that parent-focused strategies may help modify accessibility-related practices. However, the magnitude and consistency of accessibility changes required to produce meaningful improvements in diet quality warrant further investigation.

Conceptual and measurement issues surrounding accessibility should be considered. There is a need for HFE research to uniformly define accessibility in order to facilitate comparisons across studies and to better identify which aspects of accessibility influence dietary intake [10]. Interventions targeting the HFE have often measured different components of accessibility by only measuring the location but not the form (e.g., ready-to-eat) [14] or combining availability with accessibility [30]. These factors are particularly important when considering young children who may not have the ability and skills to make FVs accessible without a parent. Without assessing whether foods are prepared (e.g., washed, cut, and cooked), within reach, and visually prominent, it is difficult to determine whether true accessibility, rather than simple presence, has been achieved. In the present study, the HFI was adapted to include additional dimensions of accessibility to better capture factors influencing dietary intake. While these items were informed by prior literature [10], formal psychometric testing of the modified indicators was not conducted. Thus, findings related to these expanded accessibility measures should be considered carefully, and future research is needed to evaluate their reliability and validity.

Even though the trend of summed availability of FVs decreased from pre- to post-intervention, a slight, non-significant increase was observed only in the form (ready-to-eat) accessibility domain. This pattern suggests that certain domains of accessibility might be more important to target than others. Because availability and accessibility were assessed at a single time point, we were unable to determine whether the observed decrease in availability, including the significant reduction in fresh fruit availability, reflects true changes in household FV stocking patterns. In addition, these changes were not reflected in FV intake, which showed minimal change post-intervention. Further studies incorporating repeated or ecological momentary assessments [31] of the HFE may help clarify whether changes in availability and accessibility reflect consumption patterns over time.

The observed increase in the ready-to-eat form may also reflect the role of convenience. Prior research indicates that prewashed, pre-cut, ready-to-eat, and frozen FVs in steamable containers reduce time, preparation, and clean-up demands and may facilitate selection and use in households with children [32]. Consistent with this, parents in the feasibility study underlying the current analysis identified convenience, time constraints, and concerns about spoilage as key factors influencing their ability to make FVs readily accessible in the home [9]. These findings suggest that interventions emphasizing practical strategies to reduce preparation burden may be particularly relevant when targeting accessibility and that encouraging other forms of FVs (e.g., frozen vegetables) might be a good approach to increase consumption. While individuals might have negative biases towards canned or frozen FVs [33], researchers have found that nutrient scores for FVs are similar across categories, in particular, the frozen category [34,35].

From a behavioral perspective, accessibility may function as an environmental cue rather than a short-term determinant of intake. Although HEI scores did not change significantly, exploratory analyses suggested that participants with HEI-total scores above the national reference tended to have FVs that were more often within reach and visible across accessibility domains. Notably, among parents, being in a higher diet quality category was associated with higher within-reach FV scores, which may reflect how structuring the home environment could support their FV exposure while also modeling healthy behaviors for children. Consistent with this, a systematic review found that availability, parental control of foods available, and parental modeling were associated with desirable food cognitions and consumption [36].

Similarly, greater FV visibility scores in children’s environments were observed among those with HEI-total scores above the national average, further pointing to the potential role of environmental cues in shaping choices. While these findings are exploratory and based on a small sample, they suggest that accessibility may be a potentially modifiable aspect of the HFE. Repeated exposure to visible and ready-to-eat FVs may contribute to cue-response associations and automatic selection of these foods during meals and snacks, as supported by recent evidence linking greater FV availability in the HFE with stronger FV consumption habits [37]. Future studies with larger sample sizes using validated accessibility measures may help clarify these relationships and inform strategies to optimize the HFE.

These findings should also be interpreted within a broader socio-ecological context [3]. Although the present study focused on the physical HFE, dietary behaviors are shaped by multilevel influences. A recent systematic review highlights interactions between individual, interpersonal, community, and policy environments in shaping children’s FV intake and weight-related outcomes [38]. These broader determinants, including neighborhood food access, marketing, and social norms, may help explain why modifying in-home accessibility alone was insufficient to produce measurable improvements in diet quality over the short term.

We acknowledge several limitations of this study. The accessibility domains added to the HFI were conceptually informed but not formally validated, which may limit the interpretation of related findings. Additionally, this was a small feasibility study not powered to detect changes in objective HFE or diet quality and exploratory analyses (including dichotomized HEI subgroups) may have been underpowered. Multiple comparisons across outcomes and time points also increase the risk of type I error, and findings with marginal or borderline statistical significance should be viewed as preliminary.

The lack of significant findings may also reflect characteristics of our sample. Baseline FV availability was high, and participants were generally food secure and of higher income, which may have limited the potential magnitude of change. Furthermore, the sample was predominantly female (93.3%), which limits generalizability. Findings should therefore be interpreted as reflecting female primary food purchasers rather than more diverse caregiver populations. This aligns with prior research indicating that women are more likely to assume responsibility for meal preparation and food shopping within households [39]. Finally, objective assessment of accessibility using the HFI reflects a single-timepoint snapshot of the home environment, whereas accessibility-related behaviors such as food preparation, placement, and visibility are dynamic and may vary across days and eating occasions. The intervention duration may have been insufficient for changes in accessibility to translate into measurable improvements in overall diet quality.

Taken together, these findings support the idea that accessibility is conceptually distinct from availability and may function as a supportive environmental cue that facilitates healthier choices. Although this FV accessibility intervention did not yield statistically significant improvements in objective accessibility or diet quality, parents reported an increased frequency of having FVs prepared and ready-to-eat in their home. Exploratory analyses suggested directional patterns between FV location and parent diet quality, and FV visibility and child diet quality. Future larger, longer-term studies using validated accessibility measures are needed to clarify its independent effects and inform strategies to optimize the HFE.

## 5. Conclusions

Recognizing and incorporating the domains of accessibility in the HFE may enhance understanding of how families support children’s dietary behaviors. While this FV accessibility intervention did not yield statistically significant improvements in objective accessibility or diet quality, parents’ perceptions of the accessibility domain of form (ready-to-eat FVs) increased. Exploratory analyses suggested directional patterns linking FV location and visibility scores with diet quality among parents and children with HEI-total scores above the national average; however, these findings are exploratory in nature. The sample was predominantly female and of higher socioeconomic status, which may limit generalizability to male caregivers or more diverse populations. Overall, these results contribute to a growing body of evidence that accessibility may be conceptually distinct from availability and could represent a modifiable component of the HFE. Taken together with prior intervention research, the results support further investigation of accessibility as a complementary strategy within family-based approaches in larger, adequately powered studies. The potential impact of this intervention remains untested, given the pilot design and sample size limitations, and future work is needed to determine how targeting accessibility may influence dietary behaviors.

## Figures and Tables

**Table 1:** Demographic characteristics of FV accessibility intervention (*n* = 30).

**Parent Characteristics**	***n***	**(%)**
Sex		
Male	2	6.7
Female	28	93.3
Race		
White	21	70
African American	5	16.7
Asian	2	6.7
More than one race/mixed	2	6.7
Ethnicity		
Hispanic or Latino	3	10
Not Hispanic or Latino	27	90
Marital Status		
Married or living with a significant other	24	80
Widowed	1	3.3
Never married/single	5	16.6
BMI Categories		
Underweight/Normal weight	6	20
Overweight	10	33.3
Obesity	14	46.7
**Child Characteristics**	***n***	**(%)**
Sex		
Male	16	53.3
Female	14	46.7
Race		
White	21	70
African American	5	16.7
Asian	1	3.3
More than one race/mixed	3	10
Ethnicity		
Hispanic or Latino	3	10
Not Hispanic or Latino	27	90
BMI Percentile Categories		
Underweight/Normal weight	13	43.3
Overweight	9	30
Obesity	8	26.7
**Household Characteristics**	***n***	**(%)**
Annual Household Income		
≤$39,999	2	6.6
$40,000–$74,999	5	16.6
$75,000–$99,999	2	6.7
≥100,000	21	70.1

**Table 2:** Changes in frequency of perceived FV accessibility over the past 7 days (*n* = 30).

Measure	Response Option	Pre-Intervention (*n*)	Post-Intervention (*n*)
FVs on the kitchen counter or somewhere in the open	Seldom/never	4	2
	Some of the time	9	8
	Most of the time	16	20
Ready-to-eat fruit or ready-to-eat vegetables on the front shelf of the refrigerator that could be visibly seen when the door is open	Seldom/never	7	2
	Some of the time	9	8
	Most of the time	14	20
FVs in the refrigerator or freezer prepared so they could be readily used ^1^	Seldom/never	6	1
	Some of the time	11	10
	Most of the time	12	18

^1^ (*n* = 29).

## Data Availability

The original contributions presented in this study are included in the article. Further inquiries can be directed to the corresponding author.

## Abbreviations

The following abbreviations are used in this manuscript:

FV	Fruit and Vegetable
HEI	Healthy Eating Index
HFE	Home Food Environment
HFI	Home Food Inventory
